# Jump now, pay later: saltatory behavior trades off with body mass and facilitates shelter repair in a seed-dwelling insect

**DOI:** 10.1007/s00114-026-02110-6

**Published:** 2026-05-21

**Authors:** Kalina Palas, Meharin Nusrath, Doris L. Tung, Michela Volpe, Alexandra M. Martin, Lindsey Swierk

**Affiliations:** 1 Department of Biological Sciences, Binghamton University, State University of New York, Binghamton, NY 13902 USA; 2 Amazon Conservatory for Tropical Studies, Iquitos, Loreto, 16001 Perú

**Keywords:** Endophagy, Energy allocation, Immobilization, Lepidoptera, Mobility, Seed parasite, *Sebastiania pavoniana*

## Abstract

Many animals inhabit spatially confined shelters that provide protection but impose sensory and energetic constraints. In the Mexican jumping bean moth, *Cydia saltitans*, larvae use saltatory (“jumping”) movements to relocate their shelters (i.e., parasitized seeds) away from aversive stimuli, potentially at a high energetic cost. We tested whether these movements trade off with larval energetic reserves, development, and shelter repair. We placed larvae in one of two treatments: a control group allowed to move freely, and a stationary group in which shelter movement was physically prevented, and we quantified seed wall repair time following induced damage, larval body mass as a proxy for fat reserves, and head capsule width as a developmental stage proxy. Stationary larvae exhibited significantly higher body mass than control larvae, suggesting an energetic cost to saltatory behavior, although developmental stage did not differ between treatments. However, control larvae repaired shelter damage significantly more quickly than stationary individuals. Free movement might enable larvae to optimize positioning and leverage for repair, highlighting another adaptive function of saltatory movements, or might better promote hormone levels that support silk production for repair. Our findings highlight how individuals may prioritize energy allocation for movement, despite the energetic costs, in spatially confined and resource-limited systems. Understanding this balance provides broader insight into the evolution of behavioral and physiological strategies in species adapted to spatial or energetic constraints.

**Supplementary Information:**

The online version contains supplementary material available at 10.1007/s00114-026-02110-6.

## Introduction

Many animals take advantage of spatially confined shelters like the interiors of seeds, galls, or shells for protection during particularly vulnerable life stages (e.g., Mani 1964; Shorthouse et al. 2005; Bailey et al. 2009; Cornelissen et al. 2015; Jones et al. 2002). Animals using these spatially confined shelters can be considered secondary colonizers of microhabitats generated by ecosystem engineers, which create structures that persist beyond their own immediate use and are exploited by other species (Jones et al. 1994; 1997). In many instances, use of spatially confined shelters is an obligate part of a species’ life cycle (Tooker and Giron 2020). While they offer protection, spatially confined shelters also pose challenges, including restricted sensory input, finite internal resources, and a limited ability to escape a shelter’s environmental surroundings if needed; consequently, a variety of specialized adaptations have evolved to allow shelter-confined species to mitigate these challenges (e.g., Summers et al. 2024; Bose et al. 2020; Farley et al. 2019; Boivin et al. 2015).

Saltatory locomotion (i.e., “jumping”) is one such adaptation, demonstrated by several insect larvae that develop within spatially confined shelters. These movements move not only a larva, but also its encasing shelter, out of unfavorable environments (e.g., Manier and Deamer, 2014; Saeki et al. 2015; Humphreys and Darling 2013) or away from potential parasites or predators (Saeki et al. 2015; Day 1970). Saltatory movements are undoubtedly critical to larval survival and fitness, despite the potential physiological costs. Larvae in these confined shelters typically rely on fixed food resources, and their near-constant saltatory movements may require substantial energetic investment, as initially proposed by Janse (1920). While the energetic costs of jumping in some shelter-confined species are considered minimal (Manier and Deamer, 2014), this is equivocal (Saeki et al. 2015) and is not well understood. Shelter-confined, resource-limited larvae are therefore a fascinating group in which to study the tradeoffs of a fitness-relevant behavior and other important life processes, such as growth or recovery from damage.

Physical damage or injury to an animal is a common occurrence and triggers a cascade of physiological responses, including activation of the cellular stress response, a well-conserved network that supports cellular repair, regulates metabolic activity, and mobilizes energy reserves essential for survival (Rennolds and Bely 2023). As a result, the increased demand for energy during repair must balance with other life processes involving growth, reproduction, and predator avoidance (Rennolds and Bely 2023; Sparrow and Newell 1998). This also applies to repairs to an organism’s associated structures: for example, trap-building predators like antlions or web-building spiders must decide whether to repair or abandon their damaged structures, decisions that depend on energetic state, environmental conditions, and their expected future returns (e.g., Chmiel et al. 2000; Klokočovnik et al. 2025). Individuals behaviorally and physiologically compensate for damage to structures, regardless of whether a species produces its own structure (Day et al. 2000; Geller 1990; Watabe 1983) or maintains a structure sourced from another organism (Alcaraz and Jofre 2017; Alcaraz and Kruesi 2012; Kutsukake et al. 2009; Pike and Foster 2004). Across systems, structural damage can result in a reallocation of energy for behavioral and physiological repair responses, as maintaining or rebuilding protective structures can be energetically costly and may reduce investment in other processes such as reproduction, movement, or foraging (e.g., birds and crustaceans; McClintock 1985; Taylor et al. 2024). This emphasizes the likelihood of important tradeoffs among growth, repair, and other behaviors.

**Fig. 1:**
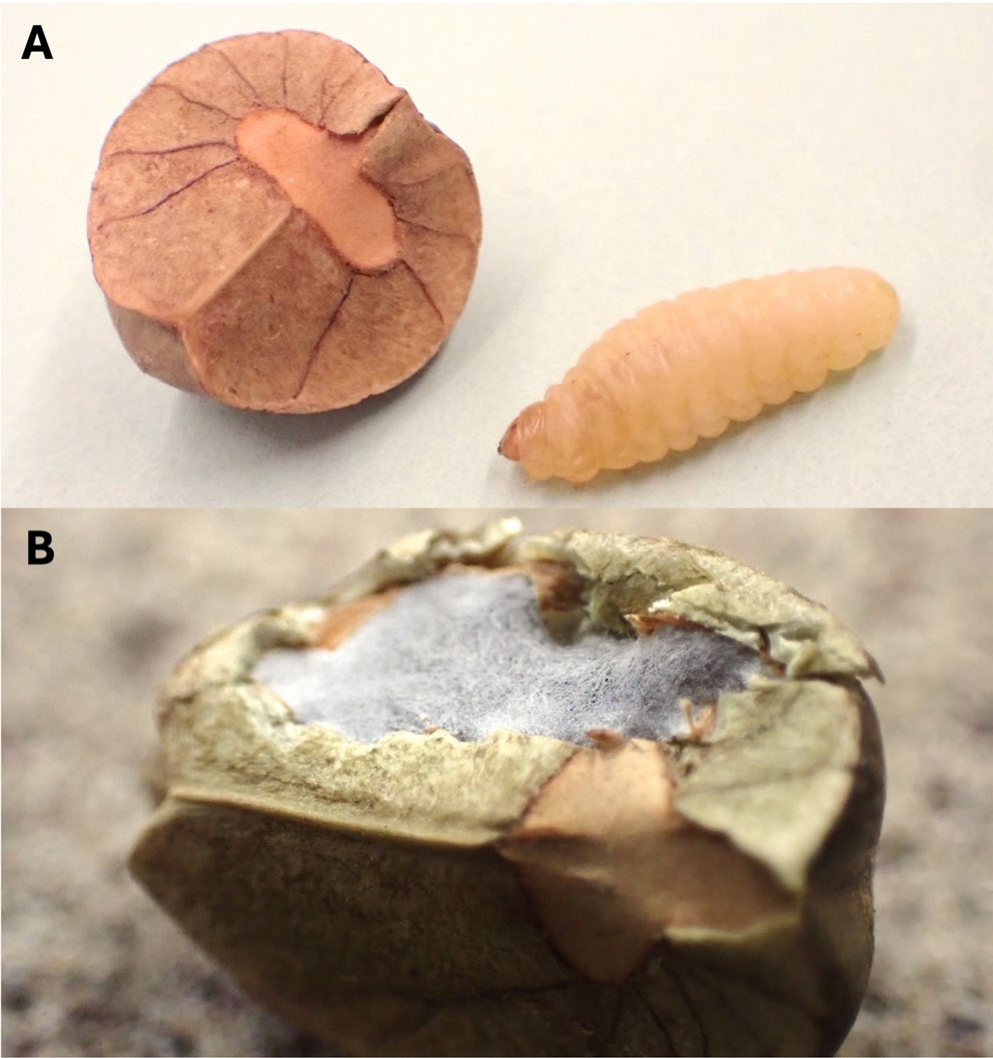
(**a**) *Sebastiania pavoniana* (host seed, left) with typical *Cydia saltitans* larva (right), and (**b**) example of *C. saltitans* silk repair following major damage to a shelter wall. Average seed length along the longest axis is ca. 9.5 mm. (Photos by L. Swierk)

The larvae of the Mexican jumping bean moth, *Cydia saltitans*, provide an intriguing model for studying the costs of near-constant saltatory movements with respect to growth and shelter repair. Native to the dry woodlands of Mexico, *C. saltitans* spend their larval phase entirely confined within seeds of *Sebastiania pavoniana* (Westwood 1858) (Fig. 1a), where they can experience extreme temperatures when exposed to sunlight. Starting in late spring, larvae spend eight or more months within a seed prior to pupation and, later, emergence (Heckrotte 1983). To mitigate risk of desiccation, larvae use saltatory movements that are thought to function, at least in part, to reposition their sheltering seed away from direct sunlight and heat (Heckrotte 1983; Gregory 1971; McKee and Tabatabai 2023). Jumping behavior in *C. saltitans* occurs through a larva’s grasping and striking behavior that moves the entire seed complex (Herter 1955; West et al. 2012). Jumping may be energetically costly, as suggested by the reduction in saltatory behavior following seed repair (Purtell 2024). Seed repair likely carries its own costs, as *C. saltitans* produces silk to patch holes or weaknesses (Fig. 1b; Supplementary Video 1), and research in other Lepidopteran larvae suggests that silk synthesis draws on fat reserves and may trade off with future reproduction (Arrese and Soulages 2010; Lye et al. 2024; but see Ruggiero and Merchant 1986).

Here, we test the hypothesis that the near-constant saltatory movements in shelter-confined *C. saltitans* larvae result in tradeoffs with larval shelter repair behaviors, growth, and development. Specifically, we predicted that larvae in an immobilized shelter (and thus spared the energetic cost of jumping) would repair seed damage more efficiently, maintain greater mass, and have faster development than those larvae allowed to move their shelters freely. Through these experiments, we aim to shed light on the broader principles of energetic tradeoffs in organisms living in resource-limited and constrained environments.

## Methods

### Animal husbandry

We purchased *Cydia saltitans* larvae in *Sebastiania pavoniana* seeds (“seed complexes”) that were collected from sites in Sonora and Sinaloa, Mexico by a commercial supplier (Amazing Beans©, amazingbeans.com; Littleton, CO, USA). Larvae were acclimated to lab conditions for 5 days in glass terraria with sand substrate (Sakrete, All-Natural Play Sand, U.S.A) and exposed to indirect natural light cycles in an enclosed laboratory space held at 21 °C. We lightly misted seed complexes twice weekly with distilled water.

## Shelter repair

Starting two weeks prior to the experiment, seed complexes were misted four times weekly due to a change in lab humidity. After initial acclimation, seed complexes were transferred to 16 plastic containers (37.9 × 38.9 × 19.3 cm, L x W x H) with sand substrate, in groups of 5. In October 2024, we conducted trials in the containers the seed complexes were housed in, eight containers per treatment group (*n* = 40 seed complexes per group). Containers were randomly assigned to the “stationary” group, in which seed complexes were immobilized, or the “free-moving control” group, in which seed complexes were allowed to freely move. A plastic grid of five 15 × 15 mm cells was pushed into non-toxic polymer clay (Sculpey Products, Stockbridge, GA, USA), as a base in the stationary treatment containers, and seed complexes were placed inside (one per cell) and likewise pushed gently 2–3 mm into the clay to immobilize them. Control group seed complexes were placed on top of clay but allowed free movement. We numbered each seed complex on the septicidal face for identification with non-toxic ink. The seed complexes were only handled during initial placement and during seed puncturing (see below), as well as for larvae retrieval from the dissected seed complex at the conclusion of the experiment.

**Fig. 2:**
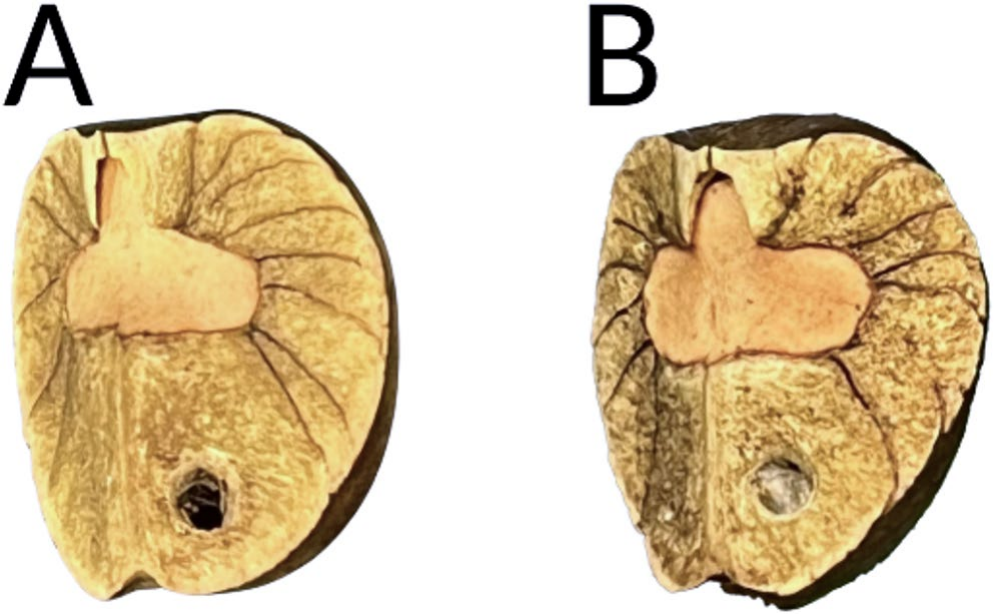
*Cydia saltitans-Sebastiania pavoniana* seed complex **(a)** immediately after experimental hole puncture on the septicidal seed wall and (**b**) with the first silk layer formed.

To begin a trial, we gently pressed a metal screw into the seed complex’s septicidal face to create a single hole at an upwards angle (to avoid any contact with larvae). Screws consistently created holes with diameters of ca. 3.7 mm (Fig. 2a). This puncturing method was used to create minimal damage similar to minor gnawing by rodents, as described by Purtell et al. (2024). After puncturing a seed complex, we returned it to its original position in the container (with stationary group seed complexes pressed back into the clay substrate), with the punctured septicidal face facing up. We used a stopwatch to track each seed complex from the initial puncture of the seed to the appearance of the first layer of silk (Fig. 2b). Punctured seed complexes were monitored continually; the first silk layer was determined to be complete when it was no longer possible to see though the hole and into the complex, either by eye or with a magnifying glass. Following trials, we extracted the larvae from the seed and recorded larval mass.

## Larval morphology and development

In November 2024, an additional set of *C. saltitans* was purchased from the same commercial supplier (Amazing Beans©, amazingbeans.com; Littleton, CO, USA) from the same sites in Sonora and Sinaloa, Mexico to examine the effects of immobilization on larval morphology and development. These seed complexes (*n* = 80) were divided randomly into two groups, as above: “stationary” and “free-moving control” (*n* = 40 per group). Seed complexes in the stationary group were pushed gently 2–3 mm into a clay substrate within individual 15 × 15 mm plastic grid cells, and those in the control group were placed on top of the clay substrate, with each seed complex individually separated by a plastic grid, as described above. Seed complexes in the control group could freely move within their allotted space, including jumping, rolling, and rotating behaviors. For the next four weeks, seed complexes were misted with distilled water twice weekly and were maintained on a 12:12 light cycle in a windowless laboratory room.

At the end of four weeks, larvae were dissected from the seed. We recorded mass to the nearest mg as a continuous measure of lipid reserves and overall nutrition (e.g., Enriquez et al. 2022) and then photographed the head of each larva under a microscope (AmScope, Irvine, California, USA) next to a calibration slide. We used ImageJ (Schneider et al. 2012) to measure head capsule width (in mm) as a comparison of development among larvae. Head capsule width (i.e., the distance between the lateral edges; Sukovata 2019; Shaheen et al. 2024) is a measurement of a sclerotized structure that changes geometrically between consecutive instars (Craig 1975).

### Statistical analysis

We ran two linear models to test the effect of treatment (stationary or free-moving control) on repair duration and the width of the head capsule. Each model contained larval mass as a covariate.

We tested the effect of treatment on larval mass using data from both experiments (“repair behavior” and “larval morphology”, see above). In this linear model, experiment was included as a factor to account for the advanced age (by one month) of larvae in the larval morphology experiment.

We used the *car* package (Fox and Weisberg 2019) to obtain *P*-values for each predictor using the Anova function. For any significant predictor, we then used the *emmeans* package (Lenth 2025) to determine the estimated marginal means of treatment on repair duration, and the *effectsize* package (Ben-Shachar et al. 2020) to calculate standardized regression coefficients as measure of effect sizes of predictors.

Model residuals were normally distributed, and model suitability was confirmed using diagnostic plots (Zuur et al. 2010). All assumptions for linear models were met. Tests were two-tailed, and alpha set to 0.05. All analyses were performed in R (R v4.3.2, R Core Team, 2023).

## Results

In the shelter repair experiment, a total of 80 seed complexes (control = 40, stationary = 40) were monitored following damage. One individual in the control group had an unusually long repair duration of 3 h, and so it was removed from analysis as an outlier (Grubb’s test, G = 6.46, U = 0.48, *P* < 0.001; *outliers* package, Komsta 2022). The effect of treatment on repair duration did not qualitatively differ with or without the inclusion of this outlier.

**Fig. 3:**
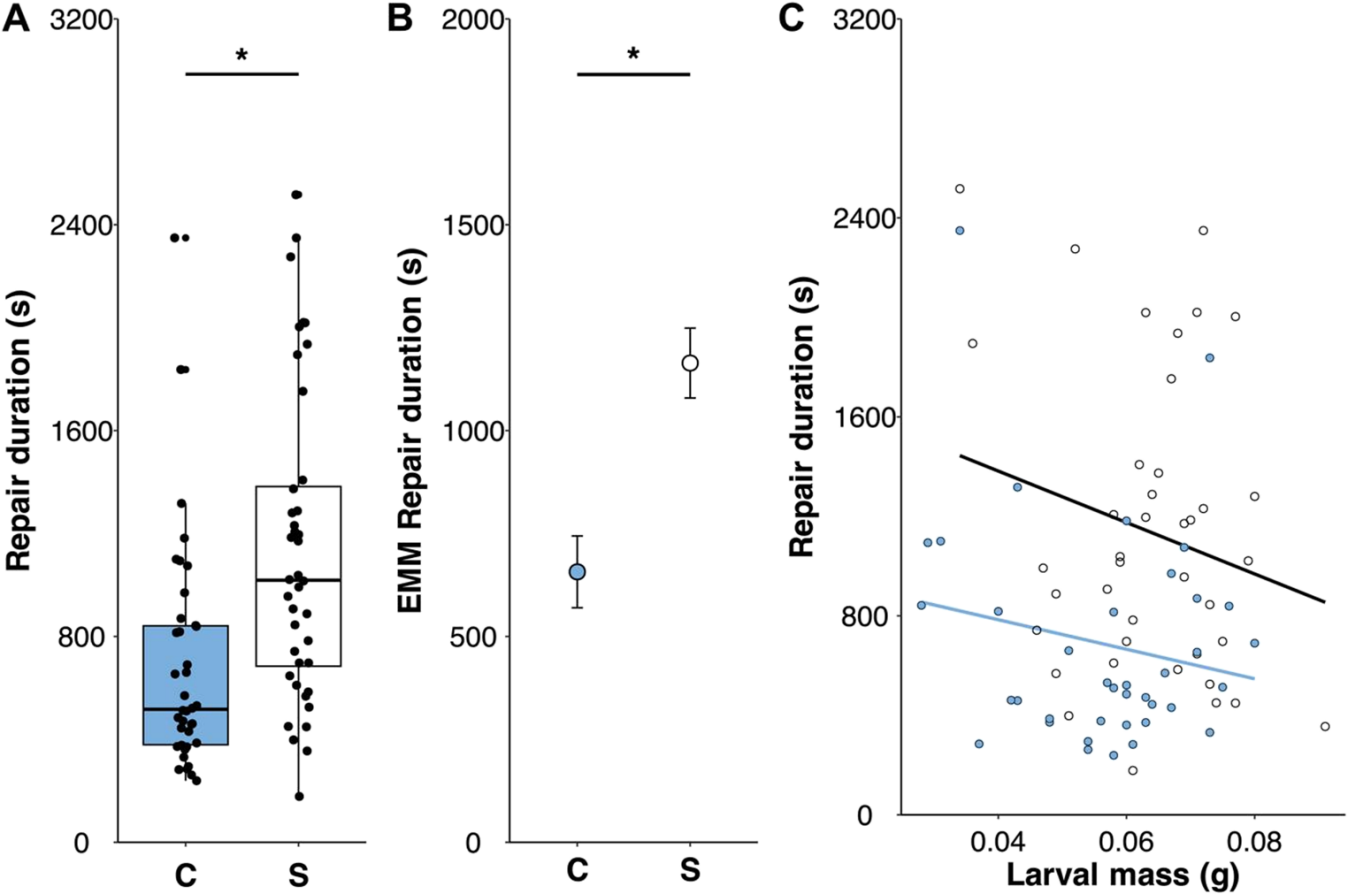
Effect of immobilization treatment, stationary (S) vs. control (i.e., free-moving; C), on (**a**) repair duration (s), (**b**) the estimated marginal means of treatment on repair duration, and (**c**) the relationships of larval mass and repair duration (S: black line, open circles; C: blue line and filled circles) of *Cydia saltitans* larvae encased in host seeds. In (**a**), median is represented by the solid horizontal line along with standard quartiles and, in (**b**), error bars represent ± 1 standard error.

Treatment significantly impacted repair duration (*F*_1,75​_ = 16.712, *P* < 0.001), such that larvae in the stationary treatment took longer to repair holes than those in the control group (Fig. 3a); estimated marginal means for repair in the stationary treatment were nearly double that of the control group (Fig. 3b), and treatment had a large effect on repair duration (standardized β = 0.88); larval mass was not correlated with repair duration (*F*_1,75​_ = 2.820, *P* = 0.097; Fig. 3c).

**Fig. 4:**
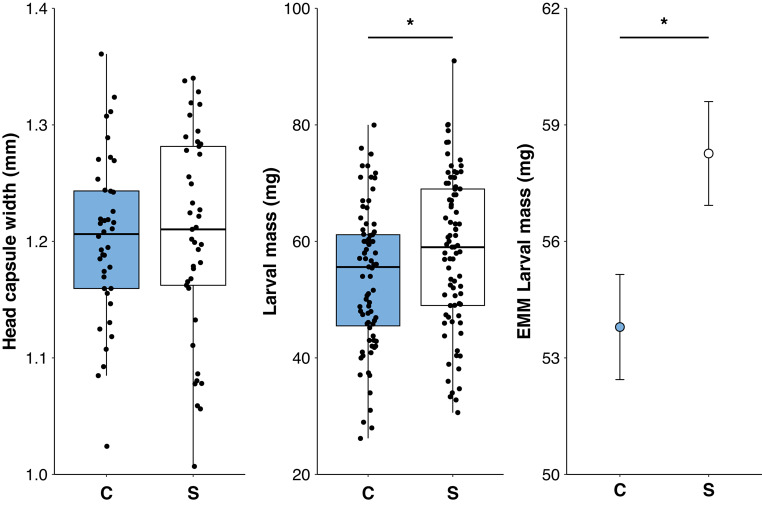
Effect of immobilization treatment, stationary (S) vs. control (i.e., free-moving; C), on (**a**) head capsule width (mm) and (**b**) body mass (mg), with (**c**) the estimated marginal means of treatment on body mass of *Cydia saltitans* larvae encased in host seeds. In (**a**, **b**), median is represented by the solid horizontal line along with standard quartiles and, in (**c**), error bars represent ± 1 standard error.

Treatment significantly affected larval mass (*F*_1,157​_ = 5.519, *P* = 0.020; Fig. 4c), with larvae in the stationary group having greater mass than those in the control group (also shown as estimated marginal means, Fig. 4d). Treatment had a medium effect on larval mass (standardized β = 0.35). Experiment also affected larval mass (*F*_1,157​_ = 17.077, *P* < 0.001). In contrast, width of the head capsule (*F*_1,78​_ = 0.024, *P* = 0.878; Fig. 4a) was not affected by treatment but was positively related to larval mass (*F*_1,78​_ = 37.771, *P* < 0.001).

## Discussion

Our results only partially agree with our original hypothesis that restricting saltatory movements in *Cydia saltitans* larvae facilitates greater larval growth along with faster shelter repair. Larvae in the stationary treatment group had greater body mass than those in the control (free-moving) group, suggesting a measurable energetic cost associated with saltatory movements for shelter relocation. However, we unexpectedly found that larvae in the stationary treatment were slower to repair shelter (i.e., seed wall) damage than those in the control group. No differences in developmental stage (using head capsule width as a proxy) were found between treatments over the four-week experiment.

The finding that stationary larvae had greater body mass than larvae in the free-moving control group is consistent with the idea that saltatory locomotion is energetically expensive: the muscular contraction and silk anchoring mechanisms required to achieve movement of the entire seed likely demand significant metabolic investment. This corresponds with evidence from other jumping larvae, in which movement trades off with energetic reserves or physiological performance (e.g., Saeki et al. 2015). Importantly, glycogen stores in larval insects are known to influence adult size and fecundity (Shin et al. 2012), suggesting that energy storage during larval stages may provide long-term fitness benefits. These energetic costs of saltatory movements may underlie their observed tradeoff with growth and also formed the basis for our initial expectations of movement’s tradeoffs with repair and development.

Our initial prediction that shelter immobilization would facilitate faster repair was based on the idea that sparing larvae the energetic cost of shelter movement (jumping) would allow them to more directly allocate resources to repair. A previous study indicated that minor damage to *C. saltitans*’s seed shelter physiologically impacts a larva’s ability to move a shelter efficiently away from an aversive stimulus, suggesting that larvae must balance seed wall repair with fitness-relevant shelter repositioning (Purtell et al. 2024). However, the longer repair times (mean = 302 s) in the stationary treatment suggest that the ability of a larva to reposition its shelter may actually be important to executing repair behaviors. One possible explanation is that free movement allows a larva to better orient itself within a shelter, providing easier access to damaged areas. That said, we observed few larval movements in the free-moving group immediately prior to shelter repair, and all larvae regardless of treatment were initially placed with the punctured septicidal face upwards: any shelter repositioning via jumping that is important to shelter repair is therefore likely to be minor.

An alternative idea is that the ability to move a shelter enables larvae to have easier access to the resources within seeds: however, while larvae may rely on typical movement patterns to access and consume internal resources of the seed, inspection of host seeds after larval dissection confirmed that all host seed material was already consumed, regardless of treatment group. In a separate study, we failed to find any consumable host seed material remaining inside seeds even prior to the dates of these experiments (early September) (Swierk, unpubl. data). Another plausible explanation of this unexpected pattern may relate to hormonal changes in larval insects: with increased levels of ecdysone (a “molting hormone”; Yamanaka et al. 2013), silk glands, which are essential to seed repair, can experience decreased function (Sehnal and Akai 1990). This pattern may be explained if jumping itself stimulates metabolic processes that alter this hormone, or if the greater body mass of the stationary larvae corresponds to increased (near-molting) levels of ecdysone.

While we observed that body mass was greater in the stationary group, suggesting that saltatory movements overall do impose a real energetic cost in these shelter-confined species, we also found that greater body mass did not relate to faster shelter repair rates. Instead, larval energy storage likely plays an important future role in adult body size and reproduction, as it does in other invertebrates (e.g., Shin et al. 2012). Regarding shelter repair, other factors like individual metabolic efficiency or behavioral variability may play a more critical role (e.g., Cornwell 2024). For instance, more active (and potentially bolder) larvae, regardless of body size, may compensate for their energetic expenditure through more rapid repair. Repair ability may also be limited by metabolic rate, which varies among individuals and is not necessarily proportional to body size (Gregorič et al. 2015; West et al. 1997; Hulbert and Else 2000). Therefore, larger larvae may not necessarily always demonstrate faster repair.

We found no significant differences between treatments in head capsule width, a proxy for larval development as sclerotized structures change in size geometrically with instar (Craig 1975). This indicates that over the four weeks of the experiment, immobilization did not significantly impact larval stage. It remains possible that longer-term immobilization could influence development if energetic savings from reduced movement are reinvested into growth, as is suggested from our results on larval mass. Future work could track molting events or measure ecdysteroid hormone levels in stationary and free-moving groups to better understand whether immobilization affects development on the longer-term. As instar durations in *C. saltitans* are currently unknown, our experimental window may not have coincided with a molt transition. We also note that other factors beyond movement and body mass may influence larval behavior, and that laboratory environmental conditions do not perfectly mimic field conditions and may have contributed to stress or adverse physiological responses.

More broadly, our findings align with work demonstrating that animals must allocate limited energy among competing processes, including movement, growth, maintenance, and repair. Such tradeoffs are a central prediction of life-history theory and have been documented across taxa, from insects to vertebrates (Metcalfe 2025). Even outside of repair contexts, animals must balance the energetic costs of movement with other functions such as reproduction and growth (e.g., Xu et al. 2024) and sensory investment or environmental assessment, as seen in systems where enhanced sensing increases locomotor costs (e.g., MacIver et al. 2010).

Our findings highlight the interplay between environmental constraints and physiological responses by examining the energy allocation tradeoffs in organisms living in spatially confined shelters. In *C. saltitans*, saltatory movement reduces body mass of larvae that were allowed to freely jump, but this does not appear to free resources for faster shelter repair as we predicted. Therefore, saltatory movements may serve multiple functions, allowing larvae to move their shelters from aversive stimuli (Heckrotte 1983; Purtell et al. 2024) and also facilitating shelter repair through postural or hormonal changes or other behaviors. These findings have broader implications for understanding the energy allocation tradeoffs and behavioral adaptations in organisms with extended architecture and external physical structures. Our results contribute to a growing body of work suggesting that confined shelters select for specialized physiological and behavioral strategies (Weaver et al. 2020; Boisseau et al. 2017; Purtell et al. 2024). As energy budgets in such systems are inherently limited, understanding how behaviors like jumping, repair, and growth are prioritized are critical for understanding survival outcomes in natural settings. Our findings contribute to a broader understanding of how animals balance competing energetic demands, a fundamental challenge shared by diverse species.

## Supplementary Information

Below is the link to the electronic supplementary material.

Supplementary Material 1 (MOV 5.69 MB)

Supplementary Material 2 (TXT 7.20 KB)

Supplementary Material 3 (TXT 2.09 KB)

Supplementary Material 4 (TXT 5.87 KB)

## Data Availability

Data are provided within the supplementary information files.
